# Higher trait neuroticism is associated with greater fatty acid amide hydrolase binding in borderline and antisocial personality disorders

**DOI:** 10.1038/s41598-022-04789-9

**Published:** 2022-01-21

**Authors:** Nathan J. Kolla, Isabelle Boileau, R. Michael Bagby

**Affiliations:** 1 Centre for Addiction and Mental Health (CAMH), 250 College Street, Room 626, Toronto, ON M5T 1R8 Canada; 2 Department of Psychiatry, University of Toronto, Toronto, ON Canada; 3 Violence Prevention Neurobiological Research Unit, CAMH, Toronto, ON Canada; 4 Waypoint Centre for Mental Health Care, Penetanguishene, ON Canada; 5 Waypoint/University of Toronto Research Chair in Forensic Mental Health Science, Penetanguishene, ON Canada

**Keywords:** Psychology, Neural circuits

## Abstract

Borderline personality disorder (BPD) and antisocial personality disorder (ASPD) are the two most frequently diagnosed and researched DSM-5 personality disorders, and both are characterized by high levels of trait neuroticism. Fatty acid amide hydrolase (FAAH), an enzyme of the endocannabinoid system (ECS), has been linked to regulation of mood through modulation of anandamide, an endocannabinoid. We hypothesized that prefrontal cortex (PFC) FAAH binding would relate to trait neuroticism in personality disorders. Thirty-one individuals with personality disorders (20 with BPD and 11 with ASPD) completed the investigation. All participants completed the revised NEO Personality Inventory, which yields standardized scores (e.g., *T* scores) for the traits of neuroticism, openness, conscientiousness, agreeableness, and extraversion. All participants were medication free and were not utilizing illicit substances as determined by drug urinalysis. Additionally, none of the participants had a comorbid major depressive episode, bipolar disorder, psychotic disorder, or substance use disorder. Each participant underwent one [^11^C]CURB PET scan. Consistent with our hypothesis, neuroticism was positively correlated with PFC FAAH binding (*r* = 0.42, *p* = 0.021), controlling for genotype. Neuroticism was also positively correlated with dorsal putamen FAAH binding (*r* = 0.53, *p* = 0.0024), controlling for genotype. Elevated brain FAAH is an endophenotype for high neuroticism in BPD and ASPD. Novel pharmacological therapeutics that inhibit FAAH could emerge as potential new treatments for BPD and ASPD with high neuroticism.

## Introduction

Neuroticism is one of the Big Five dimensional domains of personality that encompasses individual differences in negative emotional responding to frustration, threat, or loss^1–4^. It is operationally defined by items, which, in aggregate, reflect anger, sadness, irritability, self-consciousness, vulnerability, worry, and hostility that correlate well with one another in factor analyses^1–3^. Although neuroticism is associated with manifestations of psychological illness, such as mood and anxiety disorders; adverse physical health outcomes; and poorer quality of life^1^, comparably less is known about its neurochemical substrate, especially in common mental disorders. This gap in the literature hinders development of new therapeutics that could serve to mitigate the effects of high neuroticism.

Borderline personality disorder (BPD) is a debilitating illness characterized by severe emotional dysregulation, tumultuous interpersonal relationships, and self-harming and suicidal behavior^5^. According to meta-analytic review, BPD is the personality disorder most strongly associated with neuroticism^6,7^. Additionally, longitudinal analysis points to robust associations between BPD symptomatology and neuroticism^8^. Antisocial personality disorder (ASPD) is another common condition that shares clinical similarities with BPD. Expression of neuroticism positively correlates with ASPD, especially with symptoms of anger and impulsivity^9^, although meta-analysis shows smaller effect sizes for ASPD and neuroticism than with BPD and the latter^6^. Clinically, a significant positive relationship exists between neuroticism in childhood or adolescence and adult antisocial behavior^10^. ASPD and BPD are highly comorbid^11^. Higher neuroticism scores are seen in ASPD with comorbid BPD, especially among individuals with substance use disorders^12^.

Advances in the study of endocannabinoid signaling pathways have furthered our understanding of the neurochemistry of myriad psychiatric disorders (for review, see^13^). The brain endocannabinoid system (ECS) is comprised of two main G protein-coupled cannabinoid receptors, cannabinoid receptor 1 (CB1R) and cannabinoid receptor 2 (CB2R) and two principal endogenous cannabinoids (e.g., endocannabinoids), *N*-arachidonylethanolamide (anandamide or AEA) and 2-arachidonylglycerol (2-AG). Other endocannabinoid-related signaling lipids, such as the *N*-acylethanolamides, *N*-palmitoylethanolamine and *N*-oleoylethanolamine, are also known to exist but are less well-studied^14^. In a distinctive signalling pathway, AEA and 2-AG are synthesized on demand in the post-synaptic neuron and released as required into the synaptic cleft and bind, in retrograde fashion, to pre-synaptic CB1R and CB2R located on axon terminals on the pre-synaptic neuron^15^. To terminate endocannabinoid signalling, fatty acid amide hydrolase (FAAH), an integral membrane serine hydrolase, degrades AEA in the post-synaptic neuron, while 2-AG is metabolized by the enzyme monoacylglycerol lipase in the pre-synaptic neuron^13^. CB1Rs are promiscuous in the central nervous system (CNS) with high levels in the cerebral cortex, hippocampus, amygdala, striatum, and substantia nigra, and they play functional roles in regulating mood, stress, and anxiety^16,17^. FAAH is also widely distributed in the CNS, including the cerebral cortex, hippocampal formation, amygdala, and cerebellum with its regional distribution correlated with the density of CB1Rs^18–21^. Therefore, enzymatic regulation of AEA by FAAH is able to indirectly control CB1R signaling and ostensibly modulate cognitive processes underlying mood and anxiety symptoms^22^.

Using [^11^C]CURB positron emission tomography (PET), we reported that FAAH expression was elevated in the prefrontal cortex (PFC) of BPD and that PFC FAAH density correlated with measures of anger/hostility^23^. Conversely, we also found utilizing [^11^C]CURB PET that amygdala FAAH expression was lower in the amygdala of ASPD and that impulsive behaviors were negatively associated with FAAH density in other brain regions studied^24^. These findings have enhanced our knowledge about the pathophysiology of BPD and ASPD and their symptom clusters, yet an unanswered question remains how FAAH expression may relate to personality traits such as neuroticism. There is precedent for investigating FAAH and neuroticism in other psychological disorders. For example, individuals with posttraumatic stress disorder and comorbid alcohol dependence who were wildtype for the *FAAH* gene displayed increased subjective anxiety responses to a stress challenge versus subjects with a single nucleotide polymorphism of the *FAAH* gene conferring lower levels of brain in vivo FAAH expression who reported decreased anxiety for the same stress paradigm^25^. Importantly, all analyses in this study controlled for trait neuroticism. Based on these findings, we aimed to investigate FAAH brain expression in a combined sample of BPD and ASPD participants. The most consistent finding from structural magnetic resonance imaging (sMRI) studies of trait neuroticism has been structural alterations of the PFC and its subdivisions among individuals with higher neuroticism^26–31^. Therefore, we focused on the PFC and hypothesized that trait neuroticism in BPD and ASPD would be associated with greater PFC FAAH binding, although we also explored other brain regions to test this association. It should also be noted that the association between ASPD and neuroticism is weaker than for ASPD and agreeableness^32^. Since the ECS may also be related to agreeableness^33^, and AEA-mediated signaling at CB1R, driven by oxytocin, appears to control social reward^34^, we also investigated whether the agreeableness dimension would be associated with FAAH binding in the PFC and other brain regions.

## Results

Participants’ clinical and demographic information is reported in Table 1. Information on comorbid conditions is presented in Table 2.

**Table 1:** Demographic and clinical characteristics.

Age	30.8 ± 9.6 years
Sex	18 female; 13 male
Ethnicity	20 Caucasian; 6 Asian; 2 Black; 2 Hispanic; 1 Aboriginal
Education	14.3 ± 2.3 years
Full scale IQ	100.7 ± 14.8
BMI	25.6 ± 5.2
Neuroticism *T* score	73.5 ± 7.9
Anxiety *T* score	63.5 ± 11.1
Anger *T* score	73.2 ± 7.1
Depression *T* score	71.0 ± 9.5
Self-consciousness *T* score	68.5 ± 9.9
Impulsivity *T* score	63.5 ± 11.3
Vulnerability *T* score	70.4 ± 11.0
Extraversion *T* Score	44.1 ± 12.7
Openness *T* score	55.3 ± 11.1
Agreeableness *T* score	32.0 ± 10.5
Conscientiousness *T* score	34.8 ± 13.0
Specific activity of radiotracer	3778.3 ± 1296.4 mCi/μmol
Mass injected of radiotracer	9.5 ± 0.72 mCi

**Table 2:** Comorbid psychiatric conditions.

Comorbid diagnoses in sample	BPD + ASPD participants
	*n* = 31
Major depressive disorder (%)	61.3
Dysthymic disorder (%)	0
Panic disorder (%)	9.7
Agoraphobia (%)	6.5
Specific phobia (%)	6.5
Social phobia (%)	16.1
Generalized anxiety disorder (%)	35.5
Obsessive compulsive disorder (%)	6.5
Posttraumatic stress disorder (%)	22.6
Previous alcohol use disorder (%)	25.8
Previous cannabis use disorder (%)	9.7
Previous opioid use disorder (%)	3.2
Previous sedative/hypnotic use disorder (%)	3.2
Previous stimulant use disorder (%)	3.2
Previous hallucinogen use disorder (%)	3.2
Previous polysubstance dependence (%)	3.2
Somatization disorder (%)	0
Pain disorder (%)	0
Undifferentiated somatoform disorder (%)	0
Hypochondriasis (%)	0
Body dysmorphic disorder (%)	0
Anorexia nervosa (%)	0
Bulimia nervosa (%)	0
Eating disorder not otherwise specified (%)	0
Paranoid personality disorder (%)	16.1
Schizoid personality disorder (%)	3.2
Schizotypal personality disorder (%)	0
Histrionic personality disorder (%)	0
Narcissistic personality disorder (%)	0
Avoidant personality disorder (%)	25.8
Obsessive compulsive personality disorder (%)	12.9
Dependent personality disorder (%)	9.7

### Partial correlation between PFC [^11^C]CURB λ*k*_3_ and neuroticism

**Figure 1:**
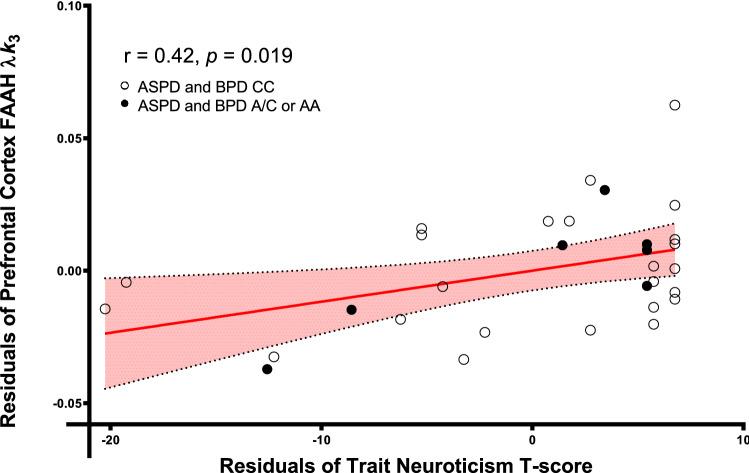
Prefrontal cortex fatty acid amide hydrolase λ*k*_3_ is correlated with trait neuroticism (*T* score) controlling for genotype in antisocial and borderline personality disorders.

We found a significant partial correlation between PFC [^11^C]CURB λ*k*_3_ and trait neuroticism, controlling for genotype (*r* = 0.42, *p* = 0.021). When we log-transformed our data, we found that the significant correlation persisted (*p* = 0.021). PFC [^11^C]CURB λ*k*_3_ was also significantly correlated with neuroticism facets anxiety (*r* = 0.42, *p* = 0.023) and self-consciousness (*r* = 0.37, *p* = 0.047), controlling for genotype, although these results were uncorrected for multiple comparisons. Figure 1 depicts the residuals of PFC [^11^C]CURB λ*k*_3_ after regressing PFC [^11^C]CURB λ*k*_3_ onto genotype by the residuals of neuroticism after regressing neuroticism onto genotype.

### Partial correlations between secondary regions of interest (ROIs) [^11^C]CURB λ*k*_3_ values and neuroticism

**Figure 2:**
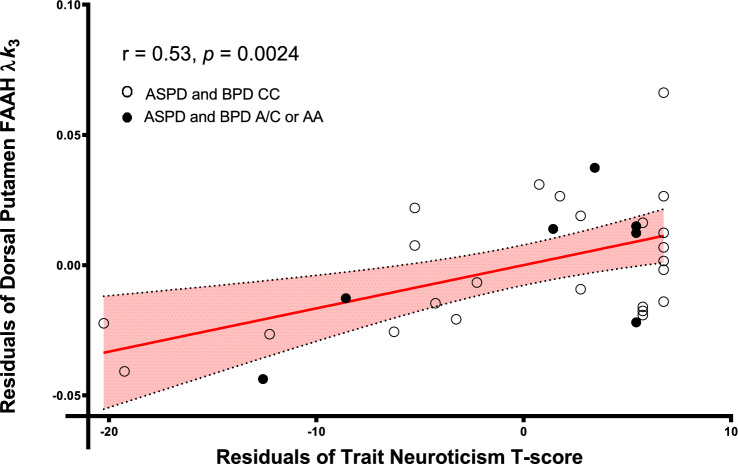
Dorsal putamen fatty acid amide hydrolase λ*k*_3_ is correlated with trait neuroticism (*T* score) controlling for genotype in antisocial and borderline personality disorders.

When we tested the relationship between [^11^C]CURB λ*k*_3_ and neuroticism in the secondary ROIs, only the relationship between dorsal putamen [^11^C]CURB λ*k*_3_ and neuroticism remained significant, after controlling for genotype and correcting for multiple comparisons (*r* = 0.53, *p* = 0.0024). Dorsal putamen [^11^C]CURB λ*k*_3_ was also positively correlated with neuroticism facets anxiety (*r* = 0.47, *p* = 0.0092), depression (*r* = 0.39, *p* = 0.034), and self-consciousness (*r* = 0.46, *p* = 0.012). These latter correlations were uncorrected for multiple comparisons. Statistics for the secondary ROIs are presented in Table 3. Figure 2 presents the residuals of dorsal putamen [^11^C]CURB λ*k*_3_ after regressing dorsal putamen [^11^C]CURB λ*k*_3_ onto genotype by the residuals of neuroticism after regressing neuroticism onto genotype.

**Table 3:** Partial correlations between trait neuroticism and secondary regions of interest, controlling for genotype.

Region	*r*	*p*-value
Anterior cingulate cortex	0.42	0.023
Temporal cortex	0.43	0.019
Hippocampus	0.40	0.030
Insula	0.48	0.0080
Thalamus	0.42	0.020
Ventral striatum	0.45	0.013
Dorsal caudate	0.45	0.013
Dorsal putamen	0.53	0.0024
Amygdala	0.46	0.010
Cerebellum	0.34	0.065

### Partial correlations between PFC [^11^C]CURB λ*k*_3_ and trait neuroticism among individuals with comorbid conditions

One of the challenges of interpreting the relationship between trait neuroticism and PFC [^11^C]CURB λ*k*_3_ is whether this finding is driven by comorbid conditions such as major depressive disorder (MDD) or anxiety disorders. For example, 61% of the sample had comorbid MDD, although none of the participants had a current major depressive episode. Similarly, 65% of the sample had a current anxiety disorder. When we examined the relationship between PFC [^11^C]CURB λ*k*_3_ and neuroticism among the participants with MDD, we did not find a significant relationship (*r* = 0.30, *p* = 0.23). However, we did detect a trend association between PFC [^11^C]CURB λ*k*_3_ and trait neuroticism among those with current anxiety disorders (*r* = 0.40, *p* = 0.092).

### Partial correlations between PFC [^11^C]CURB λ*k*_3_ and other Big Five dimensional domains of personality

PFC [^11^C]CURB λ*k*_3_ was not significantly correlated with *T* scores of the extraversion, openness, agreeableness, or conscientiousness domains (*p*-values ranged from 0.37 to 0.92).

### Partial correlations between secondary ROIs [^11^C]CURB λ*k*_3_ values and agreeableness

When we tested the relationship between [^11^C]CURB λ*k*_3_ and agreeableness in the secondary ROIs, none of the correlations were significant (*p*-values ranged from 0.13 to 0.99).

## Discussion

This study investigated the relationship between brain FAAH binding and neuroticism in a sample of ASPD and BPD participants. Consistent with our main hypothesis, we found that PFC [^11^C]CURB λ*k*_3_ correlated positively with trait neuroticism. Exploratory analyses determined that PFC FAAH binding was linked to greater anxiety and self-consciousness. A second main finding was that dorsal putamen [^11^C]CURB λ*k*_3_ similarly correlated positively with neuroticism. In terms of facet-level associations, dorsal putamen FAAH binding was positively associated with anxiety, depression, and self-consciousness. These findings have relevance for understanding the neurochemistry of personality traits in BPD and ASPD and offer tentative guidance for testing FAAH inhibitors in personality-disordered individuals scoring high in neuroticism.

There are very few studies that have explored in vivo measures of personality functioning in ASPD or BPD and none to our knowledge that have specifically assayed components of the ECS, apart from one functional MRI study of healthy individuals that found no relationship between neuroticism and amygdala [^11^C]CURB λ*k*_3_^35^. Here, we focused on the relationship between FAAH binding and neuroticism in personality disorders. Our findings comport well with the animal literature linking pharmacological inhibition or genetic deletion of FAAH to the rescue of depressive and anxious phenotypes^36^. According to this model, decreased FAAH activity increases brain AEA levels and stimulates anxiolytic-like responses in a CB1R-dependent manner^37^. In support of this view, CB1R activation in the prefrontal cortex, ventral hippocampus, and periaqueductal grey elicits anxiolytic-like effects^38,39^. Because maladaptive behavioral and neuroendocrine reactions to protracted stress contribute to manifestation of depressive-like behaviors^40^, it is thought that facilitation of AEA signaling may counter these effects by enhancing adaptive stress coping behaviors^41^ and diminishing neuroendocrine responses to psychological stressors^42^. Overall, this research suggests that augmentation of AEA-CB1R signaling through inhibition of FAAH activity may be an effective strategy for alleviating depressive and anxious symptomatology. By contrast, dampened AEA-CB1R circuitry through increased FAAH expression may contribute to increased levels of neuroticism, including trait anxiety and depression, which was observed in our sample of ASPD and BPD participants.

Bolstered by sMRI data revealing alterations of the PFC in trait neuroticism^26–31^, we similarly detected elevated PFC [^11^C]CURB λ*k*_3_ as a function of increased neuroticism in ASPD and BPD. The PFC, which broadly plays a role in affective processing and emotion regulation^43^, has been previously identified as an important locus for expression of facets of neuroticism in BPD. For example, Soloff and colleagues reported that trait impulsivity, one facet of neuroticism, was related to decreased serotonin-2A receptor (5-HT2AR) binding potential using [^18^F]altanserin PET in the medial frontal cortex among female BPD participants^44^. Studies have also examined the association of PFC 5-HT2R binding with trait neuroticism in healthy cohorts, with some findings showing contradictory findings that could be a reflection of differences in the neural substrates between clinical and healthy populations. One investigation found a positive correlation between frontolimbic 5-HT2AR and neuroticism in healthy volunteers that was mainly driven by the anxiety and vulnerability facets^45^. In another PET study of healthy participants using the radiotracer [^11^C]WAY-100635, trait anxiety was inversely correlated with 5-HT1AR binding in the dorsolateral PFC (DLPFC)^46^. A similar negative correlation was observed between DLPFC [^11^C]WAY-100635 binding potential and trait neuroticism in another sample of healthy subjects^47^. Since prominent linkages between the ECS and serotonergic system have been described^48^, we suggest that the positive association of PFC [^11^C]CURB λ*k*_3_ with neuroticism may be partially explained by interactions of the ECS with serotonergic signaling pathways. This line of reasoning is supported by preclinical evidence showing that basal serotonergic tone is higher in the frontal cortex of FAAH knockout mice following potassium-induced depolarization^49^ and that downregulation of 5-HT2AR and 5-HT2CR occurs in the PFC^48^, perhaps as a result of higher endogeneous levels of 5-HT. These neurochemical changes were paralleled by anxiolytic-like effects. Other reports have signaled that pharmacological inhibition of FAAH leads to increased brain-derived neurotrophic factor and neurogenesis in other brain regions functionally linked to the PFC^50^. In light of this evidence, we propose that increased PFC FAAH could lead to decreased endogenous 5-HT tone in affected regions that predisposes to greater expression of neuroticism. Dual radiotracer PET experiments that simultaneously measured PFC [^11^C]CURB λ*k*_3_ and 5-HT1AR or 5-HT2AR binding potential could shed light on this question.

The association between dorsal striatum [^11^C]CURB λ*k*_3_ and neuroticism may provide a mechanism to understand impaired decision-making in personality disorders. While the involvement of the dorsal striatum in control of motor movement has been well-established, evidence further implicates the dorsal striatum and its corticostriatal network in action selection and initiation^51^. Poor decision making has been demonstrated in ASPD and BPD^52^, and poor decision making has been linked to increased neuroticism in some samples^53^. Therefore, ASPD and BPD with high neuroticism may be poor decision makers. Additionally, some sMRI investigations have also shown connections between trait neuroticism and functional alterations of the striatum^54^. Preclinical research indicates that receptors involved with striatal synaptic plasticity are crucial for striatal-based learning^55^. One investigation determined that striatal CB1R receptor deletion impaired habit learning^56^. Preclinical models further demonstrated that FAAH inhibition can preserve the structural integrity of the striatum and prevent neuronal loss following an excitotoxic lesion^57^. These results indicate a role for endocannabinoids in habit learning^55^ and suggest that higher FAAH binding associated with greater neuroticism may produce deficits resulting in impaired decision making in ASPD and BPD.

When we examined the relationship between PFC [^11^C]CURB λ*k*_3_ and neuroticism in individuals who had comorbid MDD, we did not find a significant correlation. However, a trend association emerged for the relationship between PFC [^11^C]CURB λ*k*_3_ and neuroticism among participants with current anxiety disorders, which aligns with our other finding of a significant correlation between facet anxiety, but not facet depression, and PFC [^11^C]CURB λ*k*_3_. Thus, the association between PFC FAAH binding and neuroticism may be more driven by facet-level anxiety. These results coincide with clinical observations that FAAH inhibitors have shown some benefit for treatment of anxiety disorders^58^.

Several limitations of the present study must be acknowledged. First, we combined ASPD and BPD participants to investigate them as a single group. We justified collapsing ASPD and BPD into a single group, because the conditions are both Cluster B personality disorders, both are highly comorbid with one another^11^, and both show high levels of neuroticism. In fact, there was no difference in trait levels of neuroticism between the ASPD and BPD groups (ASPD = 71.4 ± 6.9, BPD = 74.8 ± 8.3; *t* =  − 1.1, df = 29, *p* = 0.26), which suggests that neuroticism levels did not relate to gender in this sample. There is also precedent for studying Cluster B personality disorders as a single group in biological studies^59,60^. Finally, using standardized *T* scores of neuroticism for all of the correlational analyses allowed for some comparison across genders. Collapsing ASPD and BPD into one group did enable us to increase the study power. Still, our investigation was likely underpowered to detect effects for the secondary ROIs; all regions showed correlations between [^11^C]CURB λ*k*_3_ and neuroticism in the expected direction but did not survive correction for multiple comparisons. Second, as this study was cross-sectional, the design cannot inform on whether PFC [^11^C]CURB λ*k*_3_ is stable to changes in trait neuroticism over time. Third, we did not sample for serum levels of endocannabinoids, such as AEA and the endocannabinoid-related signaling lipids *N*-palmitoylethanolamine and *N*-oleoylethanolamine that may be correlated with in vivo brain levels of FAAH expression. While measuring serum endocannabinoids could provide peripheral markers of FAAH expression, no clear-cut relationship has been demonstrated between peripheral and central FAAH biomarkers^61^. A fourth limitation is that none of our BPD or ASPD participants had an active substance use disorder. We purposely excluded individuals with active substance use disorders to avoid confounds of psychoactive substances on the central marker of interest. However, individuals with BPD or ASPD are noted to have elevated rates of active substance use disorders, perhaps upwards of 70% in some samples^62^. Thus, our results may not be generalizable to the larger, more common, group of BPD and ASPD individuals with comorbid substance use disorders. Finally, since [^11^C]CURB is an irreversible tracer, λ*k*_3_ is a proxy measure of FAAH availability, as absolute quantification is not possible.

In summary, we have confirmed previous results of high neuroticism in ASPD and BPD and report that PFC and dorsal caudate [^11^C]CURB λ*k*_3_ are positively correlated with trait neuroticism. With the investigation of FAAH inhibitors for anxiety disorders and anxious endophenotypes, our data support the careful testing of these pharmacological agents in personality disorders with high neuroticism. Establishing an evidence base for novel therapeutics in personality disorders would represent a positive step forward, as authorized pharmacological treatments for ASPD and BPD are currently lacking.

## Methods

All participants provided written informed consent after all study components were fully explained to them. The Research Ethics Board of the Centre for Addiction and Mental Health (CAMH) in Toronto, Ontario, Canada, approved all procedures of this investigation. All methods were performed in accordance with the relevant guidelines and regulations.

### Participants

Thirty one participants completed this investigation: 20 patients with BPD and 11 participants with ASPD. We have previously reported neuroimaging data on a subset of these participants. However, the research questions and analyses for the present investigation were different from those of the previous studies^23,24^. BPD participants were primarily female (*n* = 18), while all ASPD subjects were male. All diagnoses were verified according to results from the Structured Clinical Interview for DSM-IV Axis II Disorders (SCID-II)^63^ and Structured Clinical Interview for DSM-IV Axis I Disorders (SCID-I)^64^ by trained raters. Additionally, BPD and ASPD diagnoses were reviewed and confirmed by a forensic psychiatrist experienced in the assessment and treatment of personality disorders (NJK).

#### BPD

BPD participants were recruited from the community and the BPD Clinic at CAMH. Exclusion criteria for the BPD participants included a current major depressive episode (MDE); history of mania, hypomania, or psychotic illness; and diagnosis of substance abuse or dependence in the past 12 months as confirmed by the SCID-I. The use of psychotropic medications or herbs in the past three months was also exclusionary. For all participants in this study, neurological illness; head trauma; positive drug screen for drugs of abuse, including cannabis, on scan and assessment days; and contraindications to safe magnetic resonance imaging (MRI) scanning also precluded participation. All BPD participants screened negative for drugs of abuse on all assessment days and MRI and PET scanning days. The urine drug screen utilized was Rapid Response™, Drugs of Abuse Test Panel (BTNX Inc., Markham, Ontario, Canada) that tests for the presence of opiates, phencyclidine, barbiturates, benzodiazepines, tricyclic antidepressants, amphetamines, tetrahydrocannabinol, and methadone.

#### ASPD

ASPD participants were recruited from the local Toronto community and correctional centers. Exclusion criteria for the ASPD participants included a current major depressive episode (MDE); history of mania, hypomania, or psychotic illness; and diagnosis of substance abuse or dependence in the past 12 months as confirmed by the SCID-I. None of the participants had used psychotropic drugs or herbs in the previous three months. All ASPD participants screened negative for drugs of abuse on all assessment days and MRI and PET scanning days using the same drug assay as for the BPD participants.

All study participants were asked to refrain from using alcohol the night before and the day of PET scanning.

### Image acquisition and analysis

Each participant completed one [^11^C]CURB PET scan at the CAMH Brain Health Imaging Centre utilizing a three-dimensional HRRT brain tomograph (CPS/Siemens, Knoxville, TN, USA). We have previously reported on the radiosynthesis of [^11^C]CURB^65^. Participants lay on their backs for the duration of the scan and wore a thermoplastic mask to reduce movement. A transmission scan was conducted followed by injection of 370 ± 40 MBq (10 ± 1 mCi) of [^11^C]CURB^66^. Brain radioactivity was calculated during sequential frames of increasing duration, and the total scan time was 60 min. Next, PET images were re-constructed using a filtered back-projection algorithm with a HANN filter at Nyquist cutoff frequency^67^. Arterial samples were continuously sampled for the first 22.5 min with an automatic blood sampling system (Model PBS-101, Veenstra Instruments, Joure, The Netherlands) after [^11^C]CURB injection. Whole blood and plasma radioactivity (1500 relative centrifugal force, 5 min) was counted using a Packard Cobra II or Wizard 2480 γ-counter (Packard Instrument Co., Meridian, CT, USA) cross-calibrated with the PET system. The concentration of parent radioligand and its metabolites was measured in each manual sample (except for the one at 15 min) as previously reported^65^. Blood-to-plasma radioactivity ratios were fit using a biexponential function and parent plasma fraction utilizing a Hill function. A dispersion- and metabolite-corrected arterial plasma input function was generated as previously described^65^.

A single standard proton-density weighted brain MRI scan was acquired for each participant (TE = 17, TR = 6000, FOV = 22 cm, matrix = 256 × 256, slice thickness = 2 mm; number of excitations = 2) on a Discovery MR750 3 T MRI scanner (General Electric, Milwaukee, WI, USA) for ROI delineation. ROIs were generated automatically using in-house software (ROMI) as previously reported^68^. Time-activity curves were acquired over 60 min. in each of the ROIs and analyzed by a two-tissue compartment model with irreversible binding to the second component. FAAH binding was quantified using the composite parameter λ*k*_3_ (λ = *K*_1_/*k*_2_)^65^.

### *FAAH* polymorphism genotyping

A single nucleotide polymorphism of the *FAAH* gene (rs324420) encompasses transversion of a cytosine residue to the nucleoside adenosine (C385A) that influences the kinetics of [^11^C]CURB. C/A and A/A genotypes show lower production of in vivo brain FAAH expression compared to those with the C/C genotype^69^. For all study participants, the *FAAH* rs324420 variant was genotyped in accordance with the manufacturer’s directions for a TaqMan SNP Genotyping assay (ID C_1897306_10; Life Technologies, Burlington, Canada) on a ViiA7 instrument (Life Technologies, Burlington, Canada) using 20 ng total genomic DNA template, Perfecta FastMix II (Quantabio, Beverly, MA, USA), in a total reaction volume of 10 μL.

### Instruments

BPD and ASPD participants completed the 240-item revised NEO Personality Inventory (NEO PI-R)^70^, which was used to assess the Big Five personality dimensions. Scores for the facets underlying neuroticism (e.g., anxiety, angry hostility, depression, self-consciousness, impulsiveness, and vulnerability) were also calculated. Each facet was a sum of eight items, and the neuroticism personality trait was the sum of the facet scores (some items were reverse coded). Participants rated the NEO PI-R items on a scale ranging from (0) strongly disagree to (4) strongly agree. The NEO PI-R domain and facet scales generally have large and mostly acceptable estimates of internal reliability and retest reliability in addition to strong convergent and discriminant validity^71^. To correct for gender and age, raw scores were converted to *T* scores using published normative data^71^. Standardized scores < 45 T are in the low range, scores between 45 and 55 *T* are in the average range, and scores > 55 *T* are in the high range^71^.

### Statistical analysis

Our first model calculated partial correlations between PFC FAAH binding and NEO-PI-R neuroticism scores controlling for *FAAH* genotype with a *p*-value < 0.05 indicating significance. We also conducted exploratory analyses between PFC FAAH expression and NEO-PI-R neuroticism facet scores controlling for *FAAH* genotype with a *p*-value < 0.05 indicating significance.

Our secondary model tested the whole brain, including anterior cingulate cortex, temporal cortex, hippocampus, insula, thalamus, ventral striatum, dorsal caudate, dorsal putamen, amygdala, and cerebellum. Partial correlations were computed between each ROI and NEO-PI-R neuroticism score, controlling for *FAAH* genotype. Bonferroni correction was applied (0.05/10 ROIs = 0.005) to correct for multiple comparisons.
